# Identification of photocrosslinking peptide ligands by mRNA display

**DOI:** 10.1038/s42004-023-00898-2

**Published:** 2023-05-31

**Authors:** Yuteng Wu, M. Teresa Bertran, Dhira Joshi, Sarah L. Maslen, Catherine Hurd, Louise J. Walport

**Affiliations:** 1 Protein-Protein Interaction Laboratory, The Francis Crick Institute, London, NW1 1AT UK; 2 Department of Chemistry, Molecular Sciences Research Hub, Imperial College London, London, W12 0BZ UK; 3 Chemical Biology, The Francis Crick Institute, London, NW1 1AT UK; 4 Proteomics, The Francis Crick Institute, London, NW1 1AT UK; 5 Crick-GSK Biomedical LinkLabs, GlaxoSmithKline, Gunnels Wood Road, Stevenage, SG1 2NY UK

**Keywords:** Peptides, RNA, Chemical libraries, Drug screening

## Abstract

Photoaffinity labelling is a promising method for studying protein-ligand interactions. However, obtaining a specific, efficient crosslinker can require significant optimisation. We report a modified mRNA display strategy, photocrosslinking-RaPID (XL-RaPID), and exploit its ability to accelerate the discovery of cyclic peptides that photocrosslink to a target of interest. As a proof of concept, we generated a benzophenone-containing library and applied XL-RaPID screening against a model target, the second bromodomain of BRD3. This crosslinking screening gave two optimal candidates that selectively labelled the target protein in cell lysate. Overall, this work introduces direct photocrosslinking screening as a versatile technique for identifying covalent peptide ligands from mRNA display libraries incorporating reactive warheads.

## Abstract (web summary)

mRNA display strategies such as random non-standard peptide integrated discovery (RaPID) system allow screening of large peptide libraries to identify reversible binding to a target of interest. Here, the authors develop a covalent version of this methodology, photocrosslinking-RaPID, allowing identification of peptides that efficiently covalently modify their target of choice *via* photoaffinity labelling.

## Introduction

Photoaffinity probes are powerful reagents for studying complex biological interactions. They can be used to identify new drug targets/off-target interactions^1–4^, to uncover the structure and location of drug binding sites^5,6^, for covalent protein labelling or for selective protein immobilisation^7–9^. Photoaffinity probes typically consist of a photoactivatable functionality appended to a target-selective ligand. Upon exposure to light, the photoactivatable moiety irreversibly crosslinks to the target of interest^10^. A variety of chemistries can be used for the photoactivatable moiety, including benzophenones, diazirines and aryl azides^4,11–13^. Benzophenones are frequently used, due to their stability, relatively mild activation conditions (365 nm light), and low and reversible reactivity with water^14,15^. In addition to their use in probes, amino acids containing a benzophenone, *p*-benzoyl-l-phenylalanine (*p*Bpa), or diazirines, e.g., photo-leucine and photo-methionine, have been successfully incorporated into proteins through genetic code reprogramming to study protein–protein interactions in vitro and in vivo^16–18^.

The design and development of photoaffinity ligands for a specific target is not trivial. Current methods often rely on a rational design using existing ligands and information on their target-binding mode^5,12,19,20^. This approach frequently results in low photocrosslinking efficiency, even when basing the probe on a high-affinity ligand^20,21^. A more efficient discovery approach would involve a method to directly screen large numbers of compounds for their ability to photocrosslink to a target of interest. In vitro display technologies such as mRNA display and phage display are ideal for this purpose as they allow the generation and screening of large, randomised libraries of peptides incorporating unnatural side-chain chemistries^22^. For example, such libraries have recently been applied to the development of photoswitchable ligands through the incorporation of an azobenzene group^23^, as well as the identification of covalent inhibitors by introducing reactive warheads such as vinyl sulfones or phenylboronic acids^24,25^. The RaPID (random non-standard peptide integrated discovery) system is particularly well suited to this due to the ability to incorporate a wide variety of unnatural chemistries into peptides using flexible in vitro translation (FIT)^22,26,27^.

**Fig. 1:**
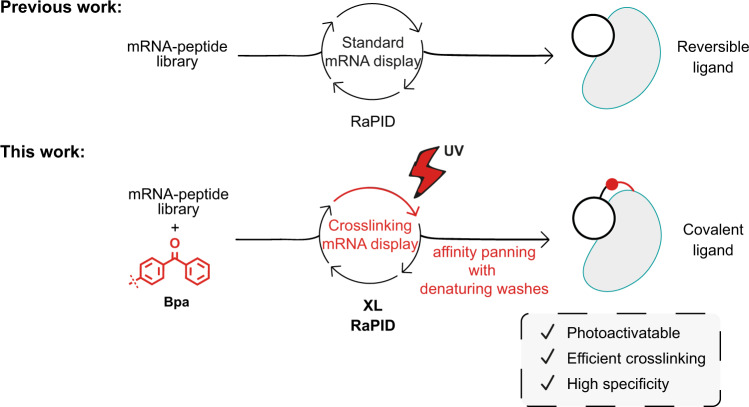
Comparison between standard and crosslinking mRNA display. Previous works describe the use of mRNA display to select reversible ligands to different proteins of interest from mRNA-displayed peptide libraries. In this work, by introducing a photocrosslinking amino acid, *p*-benzoyl-l-phenylalanine (Bpa) into peptides in the library and denaturing washes during the affinity panning selection step we describe a photocrosslinking methodology linked to mRNA display (XL-RaPID) to find macrocyclic peptides that bind covalently to a protein of interest. Modified steps are highlighted in red.

Here, we report an mRNA display strategy, photocrosslinking-RaPID (XL-RaPID), to identify peptides that undergo efficient covalent modification with their target of choice upon light activation (Fig. 1). Having first adapted the affinity panning step to isolate only covalent target-binding peptides, we applied XL-RaPID screening against a model target, the second bromodomain of BRD3 (BRD3-BD2). Our XL-RaPID screen yielded efficient photocrosslinkers that selectively labelled the target protein in cell lysate.

## Results and discussion

### Development of photocrosslinking-RaPID (XL-RaPID)

Our study began with the incorporation of the benzophenone moiety into our mRNA-displayed cyclic peptide library through including *p*Bpa in the FIT system. We synthesised the photoactivatable amino acid protected with a cyanomethyl ester (CME) group using previously reported procedures^28,29^. The CME leaving group facilitated efficient charging onto a model tRNA (microhelix RNA, FAM-MiHx_23b, Supplementary Table S1) through eFx-mediated aminoacylation (Supplementary Fig. S1). Following charging onto an elongator tRNA (tRNA^Asn^_CAU_) to genetically reprogramme the methionine codon, *p*Bpa was successfully incorporated into a model cyclic peptide by in vitro translation using a Met-free FIT system (PURExpress^TM^, Δaa, ΔtRNA, NEB) (Supplementary Fig. S2).

**Fig. 2:**
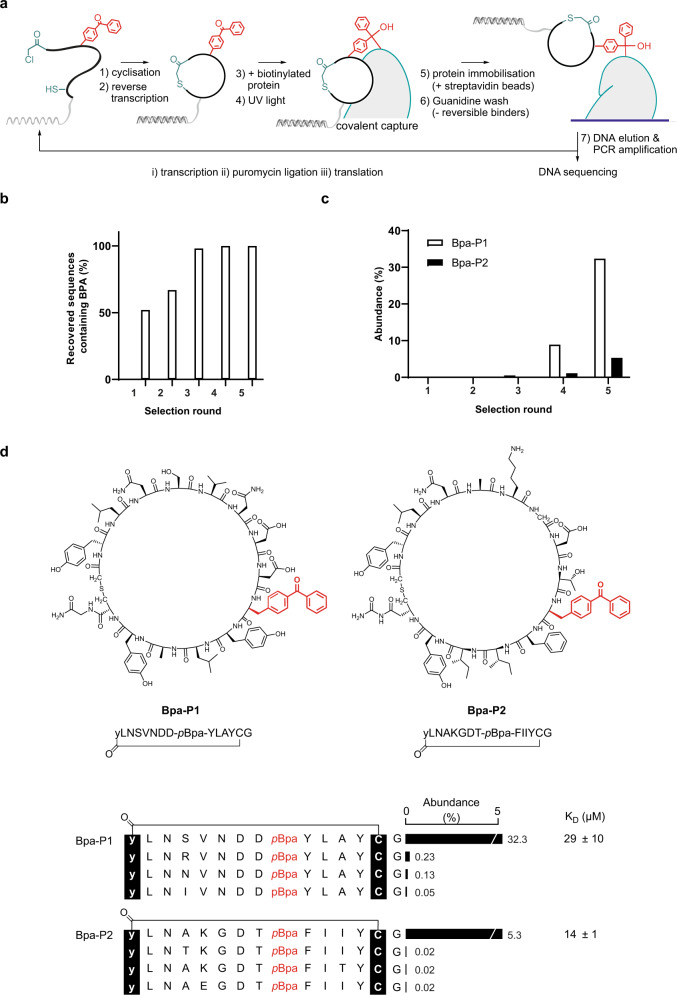
mRNA display-based selection of photocrosslinking peptide ligands. **a** Schematic depiction of the XL-RaPID selection strategy. **b** Percentage of sequences recovered after each round of selection that contains at least one *p*Bpa (calculated using the top 100 sequences). **c** Percentage abundance of the top two hits (**Bpa-P1,** **Bpa-P2**) after each round of selection. **d** Selected peptide sequences recovered after five rounds of selection. Binding affinities were determined by direct fluorescence anisotropy using FAM-labelled peptides and are represented as the mean of at least three replicates ± standard deviation.

Next, we set out to modify the standard RaPID protocol to bias it towards the selection of ligands that photocrosslink to a biotinylated target of interest. To retain only covalently cross-linked peptides during affinity panning, we envisaged replacing the standard wash buffers with wash buffers that would denature the target protein (Fig. 2a)^26^. This should allow the removal of all non-covalently bound peptides, after which the cross-linked biotinylated protein–peptide conjugates could be eluted from the streptavidin beads by boiling. In mRNA display, each peptide is covalently linked via a puromycin linker to the DNA/RNA hybrid that encoded it during translation. The DNA associated with the crosslinking peptides could therefore then be isolated, amplified by PCR and carried forward to the next round of selection. To avoid also retaining peptide sequences that crosslink to the immobilised streptavidin on the beads, or the beads themselves, we envisaged mixing the translated peptide library and biotinylated target protein and exposing them to UV irradiation to promote crosslinking in solution prior to target immobilisation on streptavidin beads.

To test the feasibility of our proposed strategy, we first confirmed that treating biotinylated protein-bound beads with guanidinium chloride (up to 8 M) did not disrupt the interaction between biotin and streptavidin (Supplementary Fig. S3). Subsequently, we used a model mRNA template encoding a previously identified non-covalent peptide ligand (3.1B, cyclic-WKTIKGKTWRTKQC(S-), K = acetylated lysine) of the first bromodomain of BRD3, BRD3-BD1^30^, to confirm the effectiveness of our denaturing washes in removing non-covalent ligands. Pleasingly, after completing a mock selection round, whilst 3.9% of the input DNA was recovered following standard affinity panning, almost no input DNA (0.0069%) was recovered following washes with 5 M guanidine (Supplementary Fig. S4). This confirmed that the guanidine washes were sufficient to dissociate a tight binding peptide (K_D_ = 0.49 nM) from its target protein^30^, most likely due to target denaturation.

Having optimised the individual steps of our XL-RaPID methodology, we set out to apply it to a model system. We aimed to identify photocrosslinking peptide ligands for the second bromodomain (BD2) of BRD3 from the bromodomain and extraterminal domain (BET) family of transcriptional coregulators^31–33^, against which we have recently identified a series of potent non-covalent cyclic peptides^30^. We constructed an mRNA-displayed peptide library (>10^12^ members) containing six to twelve random amino acids, flanked by an initiator codon and a *C*-terminal cysteine prior to a GSGSGS-linker. Peptides were initiated with *N*-chloroacetyl-d-tyrosine (by flexizyme-mediated reprogramming of the start codon), to produce macrocyclic peptides through reaction with the cysteine. Internal positions coding for methionine were reprogrammed to introduce *p*Bpa, resulting in a fraction (~28%) of the library having crosslinking capabilities. We envisaged that through iterative rounds of selection, only sequences containing at least one *p*Bpa would be enriched. The library was then used in a selection against BRD3-BD2 using our optimised XL-RaPID procedure. The peptide library and biotinylated BRD3-BD2 were first exposed to UV light (365 nm) for 30 min at 0 °C to promote crosslinking. Following protein capture by magnetic streptavidin beads, stringent 5 M guanidine washes (2 × 20 min) were used to remove ligands that were not covalently linked to the target protein. Finally, the DNA associated with the cross-linked peptides was recovered and carried forward to produce the input library for the next round of selection. We performed five rounds of selection and after observing significant enrichment in round five, next-generation sequencing was performed on the recovered DNA libraries (Supplementary Fig. S5 and Supplementary Data 1). Encouragingly, sequencing results after each round indicated a clear increase in the proportion of *p*Bpa-containing sequences in the top 100 sequences (Fig. 2b), suggesting that non-covalent ligands were effectively depleted as the selection progressed. After only the first round of selection, we observed a significant improvement to 52% of sequences containing at least one *p*Bpa, which further improved to reach 100% after round four.

Sequence alignments of the top 500 sequences from the final selection round then allowed us to identify consensus peptide sequences that were divided into distinct families. To validate the identified sequences, we selected two of the most abundant hits, **Bpa-P1,** **Bpa-P2**, which accounted for 32 and 5% of the total sequencing reads recovered in round 5, respectively (Fig. 2c, d). We synthesised both unlabelled (**Bpa-P1,** **Bpa-P2)** and fluorescently tagged (**Bpa-P1-FAM,** **Bpa-P2-FAM)** versions of these hits for analysis by a variety of assays (Supplementary Fig. S9 and Supplementary Table S2). For **Bpa-P1-FAM** and **Bpa-P2-FAM**, the 5-carboxyfluorescein label was installed at the *C*-terminus through coupling onto the sidechain of an additional lysine residue, using a selective protecting group strategy.

### Bpa-P1 and Bpa-P2 selectively photocrosslink to BRD3-BD2

**Fig. 3:**
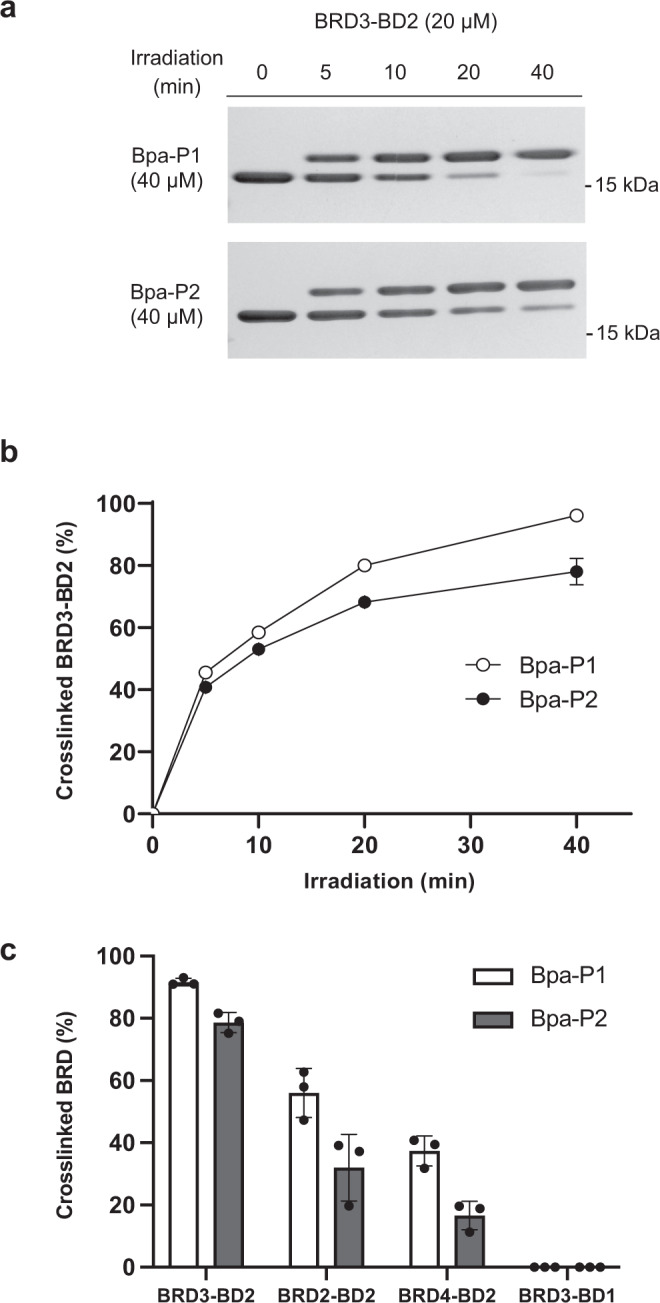
Photocrosslinking of BRDs with *p*Bpa-containing ligands. **a** Crosslinking between BRD3-BD2 (20 µM) and cyclic peptides (40 µM) after exposure to UV irradiation (365 nm) for variable amounts of time (0, 5, 10, 20, 40 min). Samples were separated on an SDS-PAGE gel and visualised by Coomassie staining. **b** Plot of percentage of cross-linked BRD3-BD2 calculated by densitometric analysis of protein bands from (**a**). Crosslinking efficiencies are represented as the mean of triplicate experiments ±1 standard deviation. **c** Crosslinking of BRD3-BD2/BRD2-BD2/BRD4-BD2/BRD3-BD1 (2 µM) by cyclic peptides (20 µM) after exposure to UV irradiation for 30 min. Samples were analysed and processed as described in (**a**, **b**). Crosslinking efficiencies are represented as the mean of triplicate experiments ±1 standard deviation.

Binding affinities for the two fluorescently tagged peptides were then determined using a direct fluorescence anisotropy assay (Fig. 2d and Supplementary Fig. S6). Interestingly, the binding affinities (**Bpa-P1-FAM**: 29 ± 10 µM, **Bpa-P2-FAM**: 14 ± 1 µM) were substantially weaker than is usually observed from a standard RaPID selection, where (sub-)nanomolar affinities are frequently observed^30,34,35^. Having established that our peptides were able to bind to BRD3-BD2, we next assessed the crosslinking efficiency of the unlabelled variants. We were pleased to find that both peptides efficiently cross-linked to BRD3-BD2 upon exposure to UV light. After incubation with two equivalents of peptide and irradiation at 365 nm for only 5 min, we observed around 40% protein modification as analysed by sample separation on an SDS-PAGE gel and Coomassie staining. As expected, longer UV exposure led to higher protein modification, reaching 96% for **Bpa-P1** and 78% for **Bpa-P2** after 40 min (Fig. 3a, b and Supplementary Fig. S10). This superior crosslinking ability of **Bpa-P1** corresponded well with its higher sequence enrichment in the selection, though it contrasts with the slightly weaker binding affinity we determined. This discrepancy may be explained by the benzophenone moiety in **Bpa-P1** being more optimally positioned for protein crosslinking than in **Bpa-P2**. Crosslinking of a given sequence to the target protein occurs in two steps. The peptide first binds reversibly to the target protein and then subsequently undergoes protein crosslinking if the benzophenone radical comes into proximity with a suitably reactive bond on the protein during its lifetime. We therefore speculate that once peptides in the library surpass a certain off-rate threshold, enrichment from the selection is based predominantly on the rate of crosslinking. Consequently, XL-RaPID is likely biased towards the identification of peptides with optimal photocrosslinking efficiency rather than tight binding affinities.

Next, we explored the specificity of our peptides by testing their ability to crosslink to other closely related bromodomains in the BET family. In addition to BRD3, the BET family consists of three other conserved members (BRD2, BRD4 and BRDT) each possessing a pair of tandem bromodomains^31,36^. Interestingly, despite their high sequence similarities, we found that the peptide ligands cross-linked more efficiently to BRD3-BD2 than to the closely related BRD2-BD2 (1.6× for Bpa-P1 and 2.0× for Bpa-P2) and BRD4-BD2 (2.8× for Bpa-P1 and 4.8× for Bpa-P2). Further, no crosslinking was observed to the *N*-terminal BD of BRD3 (BRD3-BD1) (Fig. 3c and Supplementary Fig. S11). Similar to results with BRD3-BD2, across the proteins that showed covalent modification, **Bpa-P1** cross-linked to a greater extent than **Bpa-P2**.

**Fig. 4:**
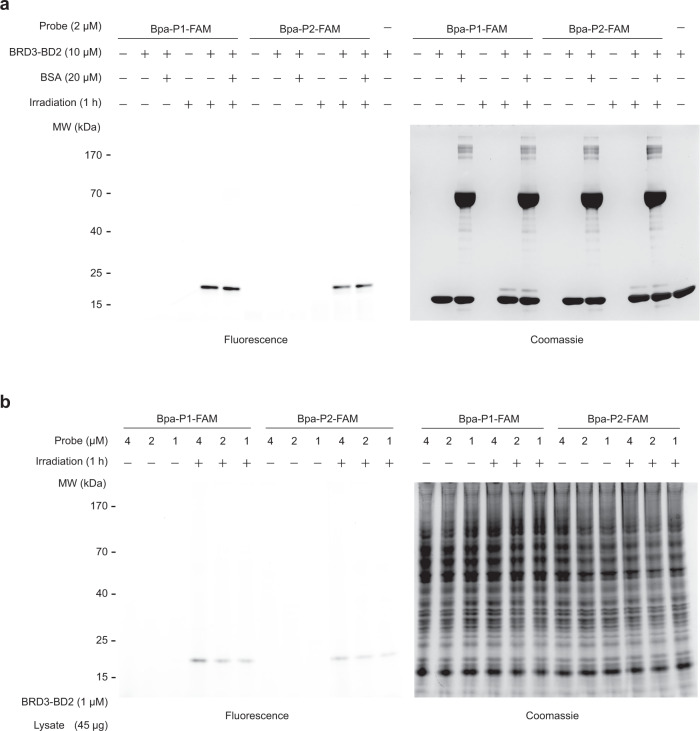
FAM cyclic peptides selectively label BRD3-BD2 in complex mixtures. **a** Mixtures containing BRD3-BD2 (10 µM, MW: 16,962) and peptide (2 µM, Bpa-P1-FAM/Bpa-P2-FAM) with/without the presence of BSA (20 µM, MW: ~66,000) were irradiated at 365 nm for 1 h. Samples were separated on an SDS-PAGE gel and visualised by in-gel fluorescence and Coomassie staining. Control experiments were performed without UV irradiation. **b** Photoaffinity labelling of BRD3-BD2 (1 µM) spiked into cell lysate (45 µg in 10 µL, HEK 293T cells). BRD3-BD2 was preferentially labelled by various concentrations of peptide (4, 2 or 1 µM) after UV exposure (1 h). Control experiments were performed without UV irradiation. Samples were analysed as described in (**a**).

To further assess the selectivity of the crosslinking ligands, we first tested whether our FAM-labelled peptides could selectively label purified recombinant BRD3-BD2 when mixed with BSA (Fig. 4a and Supplementary Fig. S12). After exposure to UV light for 1 h, both peptides selectively labelled BRD3-BD2 in the presence of BSA as observed by in-gel fluorescence of the FAM label after separation by SDS-PAGE. Control experiments were carried out without irradiation, where no labelling was observed. Next, we evaluated labelling in cell lysate (HEK 293T) that had been spiked with purified recombinant BRD3-BD2 (Fig. 4b and Supplementary Fig. S13). Pleasingly, we observed high levels of selectivity at a range of ligand concentrations (4, 2, 1 µM). Even at the highest concentration tested, we did not see any significant off-target background labelling. Consistent with our earlier experiments with the untagged peptides, these crosslinking experiments using the labelled peptides show **Bpa-P1** is a moderately better crosslinker than **Bpa-P2**. Together the data with purified proteins and in lysate suggest that our peptides are selective for their protein target.

### Bpa-P1 and Bpa-P2 photocrosslink distal to the main acetyl-lysine binding pocket of BRD3-BD2

**Fig. 5:**
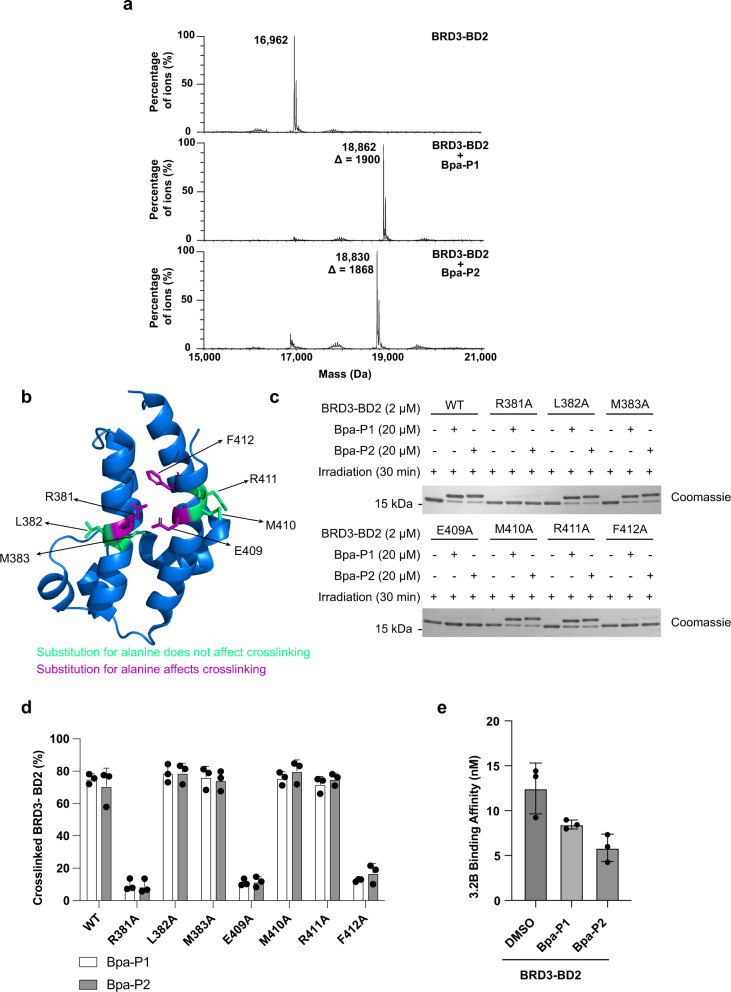
Identification of photocrosslinking site. **a** Confirmation of photocrosslinking of BRD3-BD2 by intact mass spectrometry. A mixture of BRD3-BD2 (2 µM) and cyclic peptides (20 µM) were exposed to UV irradiation for 30 min and analysed by LC–MS. The mass shifts correspond to adducts formed from the addition of one molecule of **Bpa-P1** or **Bpa-P2**. **b**, Ribbon representation of an X-ray crystallography structure of BRD3-BD2 (PDB ID: 5A7C^43^). Amino acids important for the crosslinking to **Bpa-P1** and **Bpa-P2** are shown in magenta and amino acids not required for crosslinking in green. **c** Crosslinking between wild-type or variant BRD3-BD2 (2 µM) and cyclic peptides (20 µM) after exposure to UV irradiation for 30 min. Samples were separated on an SDS-PAGE gel and visualised by Coomassie staining. **d** Quantification of (**c**) representing the mean and standard deviation of three different repeats. **e** Binding affinities for peptide 3.2B (cyclic-WSWLC(S-)KKYNLIH, K = acetylated lysine) binding to BRD3-BD2 cross-linked to **Bpa-P1** or **Bpa-P2** measured by SPR. Data represents mean ± standard deviation of the mean.

Finally, we went on to investigate the mechanism of binding of the peptides to BRD3-BD2. First, mass spectrometry (MS) was used to confirm that the protein modification observed was due to the addition of a single peptide molecule. Consistent with this, following irradiation of BRD3-BD2 with each peptide, we observed a mass shift corresponding to the addition of a single peptide (Fig. 5a). We then used limited trypsinolysis followed by MS to find the region of BRD3-BD2 to which the cyclic peptides were crosslinking (Supplementary Fig. S7). We did not observe any short peptides that could be attributed to a peptide from BRD3-BD2 covalently linked to **Bpa-P1** or **Bpa-P2**. However, while many of the shorter peptides observed were common between all three samples, we identified two peptides that were present in the non-cross-linked sample but absent when BRD3-BD2 was cross-linked to **Bpa-P1**, suggesting that the peptides could be crosslinking to the region containing amino acids 380–390 (Supplementary Fig. S7c).

To explore this further, we generated a series of BRD3-BD2 variants with single amino acid substitutions in and around this region (Fig. 5b). Crosslinking experiments with these variants demonstrated that arginine 381, glutamic acid 409 and phenylalanine 412 are essential for the crosslinking of **Bpa-P1** and **Bpa-P2** to BRD3-BD2 (Fig. 5b–d). Whilst these residues are not adjacent in the primary protein sequence, when mapped onto a crystal structure of the protein the side chains of all three residues point towards each other from two adjacent α-helices, defining a clear binding patch (Fig. 5b and Supplementary Fig. S14). Interestingly this implies the peptides bind distal to the main acetyl-lysine binding pocket with which almost all previously identified BET-BD-binding peptides and small molecules interact, suggesting that covalency may give access to new protein binding surfaces. Consistent with this model, crosslinking of BRD3-BD2 to **Bpa-P1** or **Bpa-P2**, did not block binding to a second peptide, 3.2B (cyclic-WSWLC(S-)KKYNLIH, K = acetylated lysine), previously characterised as binding to the acetyl-lysine binding pocket of the BRD3-BD2 (Fig. 5e and Supplementary Fig. S8)^27^.

## Conclusion

In conclusion, we have developed XL-RaPID, a generally applicable strategy to isolate photoactivatable ligands to a target protein of interest. Application of XL-RaPID to BRD3-BD2 resulted in *p*Bpa-containing cyclic peptide ligands that efficiently and selectively photocrosslinked with their target, even in the context of complex cell lysates. Our peptides showed substantially weaker binding affinities than is usually found in RaPID screens, suggesting that crosslinker positioning is likely more important than affinity. This highlights the power of selecting directly for photocrosslinking efficiency rather than retrofitting a crosslinking moiety to a known ligand.

In addition, our data suggest these peptides bind to a previously unliganded site on BRD3-BD2, implying that the addition of a covalent peptide-protein linkage has the potential to open up new binding space. This binding space could be exploited for protein immobilisation e.g., for biosensor development or industrial biocatalysis^37,38^, or as new druggable space. To further explore this new druggable space, peptides such as Bpa-P1 could either be converted into non-covalent binders through sequence optimisation or could be used as competitive probes in assays to identify new ligands to these new sites, either from standard RaPID libraries or from small-molecule screening libraries.

More generally, in the future, the same selection approach could be used to produce photocrosslinking ligands containing other warheads compatible with in vitro translation, such as diazirines^39–42^. Further, we envisage that this same modified affinity panning protocol could be applied more widely to the discovery of covalent cyclic peptide ligands from mRNA display libraries incorporating reactive covalent warheads.

## Methods

### Protein expression and purification

Biotinylated bromodomains, BRD2-BD2 (347–455, P25440), BRD3-BD2 (306–421, Q15059), BRD4-BD2 (348–464, O60885), BRD3-BD1 (25–144, Q15059), and all BRD3-BD2 mutants were expressed as previously described^30^. Constructs in a pQE80L-Navi vector were transformed into BL21(DE3) *Escherichia Coli* cells. A single colony was used to inoculate a LB starter culture, which was grown overnight with shaking and used to inoculate LB expression cultures (1:100 dilution). Cultures were incubated at 37 °C, with shaking at 180 rpm, until they reached mid-log phase (OD_600_ = 0.6–0.8) at which point the temperature was dropped to 18 °C and cultures were induced with 0.2 µM IPTG and 100 µM biotin. Cultures were harvested by centrifugation after ~18 h, and pellets were stored at −80 °C.

Protein pellets were resuspended in lysis buffer (5× w/v, 50 mM Tris, pH 8.0, 500 mM NaCl, 20 mM imidazole, 5 mM beta-mercaptoethanol, 0.1% triton X100) supplemented with DNase I, cOmplete EDTA-free protease inhibitor (Roche) and lysozyme. Samples were lysed by sonication and the resulting lysate was clarified by centrifugation (22k rpm, 45 min), filtered and applied to a 1 mL HisTrap HP column (GE Healthcare). The column was washed until the UV absorbance at 280 nm returned to baseline and bound protein eluted with an imidazole gradient (20–250 mM). Bromodomain-containing fractions were pooled, concentrated to <1 mL, and applied to a HiLoad® 16/600 Superdex® 75 column (GE Healthcare) pre-equilibrated with 50 mM Tris, pH 8.0, 150 mM NaCl, 1 mM DTT. Pure fractions of protein as assessed by SDS-PAGE were pooled, concentrated and snap-frozen in aliquots at −80 °C.

### Crosslinking RaPID screening (XL-RaPID)

RaPID screens were adapted from protocols previously described^30^. In brief, puromycin-ligated randomised mRNA libraries (NNK6–NNK12) were in vitro translated (30 min, 37 °C then 12 min, 25 °C) using a custom transcription/translation mixture supplemented with ClAc-D-Tyr-tRNA^fMet^_CAU_ (25 µM) and *p*Bpa-tRNA^Asn^_CAU_ (25 µM). Methionine and 10-formyl-5,6,7,8-tetrahydrofolic acid were not included in the translations. First-round translations were performed on a 150-µL scale, and subsequent rounds on a 10-µL scale. Following the addition of 200 mM EDTA (pH 8.0), the translated mixture was reverse transcribed with M-MLV RTase, RNase H minus. The resulting mixture was first buffer exchanged into selection buffer (50 mM HEPES, 150 mM NaCl, 2 mM DTT, 0.1% Tween, pH 7.5) using a 1 mL sephadex column (G-25 fine, GE Healthcare) before the addition of 2× blocking buffer (50 mM HEPES, 150 mM NaCl, 2 mM DTT, 0.1% Tween, 0.2% (w/v) acetylated bovine serum albumin, pH 7.5). Biotinylated BRD3-BD2 (200 nM) was then added, and the resulting mixture was irradiated at 365 nm in a Longwave Ultraviolet Crosslinker (model CL-1000 L, UVP, analytikjena) for 30 min at 0 °C. The irradiated mixture was then incubated with Dynabeads M-280 streptavidin (Life Technologies) for 15 min at 0 °C. The bead-immobilised protein was washed with 5 M guanidine HCl (2 × 20 min) at 0 °C before further washing with ice-cold selection buffer (3 × 1 min). PCR solution was added and the retained peptide-mRNA/DNA hybrids were eluted from the beads by heating (95 °C, 5 min). Library enrichment was assessed by quantitative real-time PCR relative to the input DNA library using primers T7g10M.F46 and CGS3an13.R22. The recovered DNA was amplified by PCR and used as the input for the next selection round.

Following five rounds of crosslinking RaPID selection, double-indexed libraries (Nextera XT indices) were prepared from recovered library DNA from rounds one to five and sequenced on the Illumina HiSeq 4000 or NovaSeq platform with single-ended 100 bp reads. Each DNA sequence was converted to a peptide sequence and ranked by total read number. For primers used to generate reagents and prepare the library for sequencing, see Supplementary Table S1.

### General crosslinking protocol

A mixture of peptide (Bpa-P1, Bpa-P2, Bpa-P1-FAM or Bpa-P2-FAM) and recombinant bromodomain (BRD3-BD2, BRD2-BD2, BRD4-BD2 or BRD3-BD1) in assay buffer (50 mM HEPES, 150 mM NaCl, 2 mM DTT, 0.1% Tween) was irradiated at 365 nm in a Longwave Ultraviolet Crosslinker (model CL-1000 L, UVP, analytikjena) for the indicated times at 0 °C. Labelling specificity experiments were performed as above with the addition of 20 µM BSA (pH 7.0, Sigma-Aldrich). Irradiated samples were analysed by SDS-PAGE or LC–MS as described in Supplementary Methods. Experiments were conducted in triplicate.

### Reporting summary

Further information on research design is available in the Nature Portfolio Reporting Summary linked to this article.

## Supplementary information

Supplementary Information

Description of Additional Supplementary Files

Supplementary Data 1

Reporting Summary

## Data Availability

Detailed Supplementary Methods and Supplementary Figs. are provided in the Supplementary Information. Sequencing data are provided in Supplementary Data 1.
