# Supplementary material for: Identification of photocrosslinking peptide ligands by mRNA display
Source: Commun Chem. 2023 May 31;6:103. doi: 10.1038/s42004-023-00898-2 (PMC10232439; doi:10.1038/s42004-023-00898-2)
Supplement: Supplementary file 1 — Supplementary Information [file 42004_2023_898_MOESM1_ESM.pdf]

# Identification of photocrosslinking peptide ligands by mRNA display

Supplementary information

Yuteng Wu, § [a,b] M. Teresa Bertran, § [a] Dhira Joshi,[c] Sarah L. Maslen,[d] Catherine Hurd,[a,e] and Louise J. Walport*[a,b]

a. Protein-Protein Interaction Laboratory, The Francis Crick Institute, London NW1 1AT, United Kingdom

b. Department of Chemistry, Molecular Sciences Research Hub, Imperial College London, London W12 0BZ, United Kingdom

c. Chemical Biology, The Francis Crick Institute, London NW1 1AT, United Kingdom

d. Proteomics, The Francis Crick Institute, London NW1 1AT, United Kingdom

e. Crick-GSK Biomedical LinkLabs, GlaxoSmithKline, Gunnels Wood Road, Stevenage, SG1 2NY, United Kingdom

*Email address: l.walport@imperial.ac.uk

§These authors contributed equally to this work

# Table of contents

## Supplementary Methods

**S1.** mRNA synthesis

**S2.** Aminoacylation of microhelix RNA and tRNA

**S3.** Test translation and MALDI-TOF mass spectrometry

**S4.** Clone assay

**S5.** Bead-protein stability

**S6.** Peptide synthesis

**S7.** Direct fluorescence anisotropy assay

**S8.** Crosslinking

**S9.** Surface plasmon resonance (SPR)

**S10.** Limited trypsinolysis

**S11.** Mutagenesis

## Supplementary Figures

**Supplementary Figure S1.** Aminoacylation efficiency

**Supplementary Figure S2.** Test translation

**Supplementary Figure S3.** Bead binding assessment

**Supplementary Figure S4.** Clone assays

**Supplementary Figure S5.** Selection recoveries

**Supplementary Figure S6.** Hit peptide binding affinities

**Supplementary Figure S7.** Limited trypsinolysis

**Supplementary Figure S8.** Surface plasmon resonance (SPR) crosslinking gels

**Supplementary Figure S9.** LCMS chromatograms of purified peptides

**Supplementary Figure S10-14.** Full SDS-PAGE gels

## Supplementary Tables

**Supplementary Table S1.** List of oligonucleotides

**Supplementary Table S2.** LCMS data for cyclic peptides

## Supplementary References

# Supplementary Methods

## S1. mRNA synthesis

### Individual Templates:

mRNA templates 1 (P0) and 2 (3.1B) were constructed by two rounds of overlapping PCR. In brief, the first round of PCR was performed at 100 µL scale (1X KOD polymerase buffer, 1 mM $MgCl_2$, 0.2 mM dNTPs, 0.6 µM T7g10M.F46 primer, 0.5 µM Primer 1 or 2, 0.8 µL KOD polymerase) for 5 cycles using an annealing temperature of 55 °C. The second round of PCR was performed at 200 µL scale using the products (20 µL) of the first round as templates (1X KOD buffer, 1 mM $MgCl_2$, 0.1 mM dNTPs, 0.25 µM T7g10M.F46 primer, 0.25 µM CGS3an13.R39 primer, 1.6 µL KOD polymerase) with an annealing temperature of 61 °C for 4 cycles. See Supplementary Table S1 for primers used in this study.

The PCR product was purified by phenol-chloroform extraction followed by ethanol precipitation. The purified product was then transcribed overnight using T7 RNA polymerase (Thermo Scientific) following the manufacturer's protocol. The RNA was isolated by isopropanol precipitation and further purified by urea denaturing 8% PAGE gel (19:1 acrylamide/bis-acrylamide).

### Library mRNA:

mRNA libraries of various length (NNK6-NNK12) were constructed individually by two rounds of overlapping PCR. In brief, the first round of PCR was performed at 100 µL scale (1X KOD polymerase buffer, 1 mM $MgCl_2$, 0.2 mM dNTPs, 0.6 µM T7g10M.F46 primer, 0.5 µM stdlib_NNK6-12, 0.8 µL KOD polymerase) for 5 cycles using an annealing temperature of 55 °C. The second round of PCR was performed at 1000 µL scale using the products (100 µL) of the first round as templates (1X KOD buffer, 1 mM $MgCl_2$, 0.1 mM dNTPs, 0.25 µM T7g10M.F46 primer, 0.25 µM CGS3an13.R39 primer, 8 µL KOD polymerase) with an annealing temperature of 61 °C for 4 cycles.

The PCR product was purified by phenol-chloroform extraction followed by ethanol precipitation. The purified product was then transcribed overnight using T7 RNA polymerase (Thermo Scientific) following the manufacturer's protocol. The RNA was isolated by isopropanol precipitation and further purified by urea denaturing 8% PAGE gel (19:1 acrylamide/bis-acrylamide).

## S2. Aminoacylation of microhelix RNA and tRNA

### Cyanomethyl ester (CME) substrates:

Aminoacylation was performed by mixing 5 mM CME substrates (ClAc-D-Tyr-CME, ClAc-L-Trp-CME or *p*Bpa-CME) with 600 mM $MgCl_2$, 20% DMSO, 25 µM eFx and either 25 µM microhelix (FAM-MiHx_23b, Integrated DNA Technologies) for determining aminoacylation efficiency or 25 µM initiator $tRNA^{fMet}_{CAU}$ (for loading ClAc-D-Tyr-CME and ClAc-L-Trp-CME) and 25 µM elongator $tRNA^{Asn}_{CAU}$ (for loading *p*Bpa-

CME), 50 mM HEPES-KOH (pH 7.5) for translations and selections. The mixture was incubated for 2 h on ice. Flexizyme eFx, initiator tRNA and elongator tRNA were synthesised according to the previously described protocol.[1] The resulting aminoacyl-microhelix/tRNA was purified by ethanol precipitation. Pellets were washed twice with 70% ethanol containing 0.1 M sodium acetate (pH 5.2), and analysed on a 20% polyacrylamide gel containing 50 mM sodium acetate (pH 5.2) by detection of the FAM label on a Typhoon FLA 9500 (GE Healthcare) and quantified with Fiji.

**Dinitrobenzyl ester (DBE) substrates:**

Aminoacylation was performed by mixing 5 mM DBE substrate (acetylated lysine, AcK-DBE) with 600 mM $MgCl_2$, 20% DMSO, 25 µM dFx, 25 µM elongator $tRNA^{Asn}_{CAU}$, 50 mM HEPES-KOH (pH 7.5). The mixture was incubated for 72 h on ice. Flexizyme dFx, was synthesised according to the previously described protocol.[1] The resulting aminoacyl-microhelix/tRNA was purified as described above for CME substrates.

## S3. Test translation and MALDI-TOF mass spectrometry

Translation of model peptide P0 was performed using a PURExpress™ Δ (aa, tRNA) *in vitro* protein synthesis kit (NEB) according to the manufacture's protocol, but with homemade solution A (50 mM HEPES-KOH, 2 mM ATP (Jena Bioscience), 2 mM GTP (Jena Bioscience), 1 mM CTP (Jena Bioscience), 1 mM UTP (Jena Bioscience), 20 mM creatine phosphate, 100 mM potassium acetate, 2 mM spermidine, 6 mM magnesium acetate, 1.5 mg/ml *E.Coli* tRNA (Roche), 14 µM DTT). Translation mixtures were prepared on ice by combining 0.78 µL solution A, 1.5 µL solution B, 0.5 µL each aminoacyl-tRNA (ClAc-D-Tyr-$tRNA^{fMet}_{CAU}$ and *p*Bpa-$tRNA^{Asn}_{CAU}$, prepared as described above and resuspended in 1 mM NaOAc), 0.5 µL mRNA template (Template 1, P0, 10 µM), 0.5 µL amino acid mixture (19 natural amino acids, methionine was excluded), 0.72 µL water. The translation reaction mixture was incubated at 37 °C for 1 h. The resulting mixture was desalted and concentrated with $ZipTip_{u\text{-}c18}$ (Millipore), co-crystallised with α-cyano-4-hydroxycinnamic acid and analysed in positive linear mode using a Micromass MALDI-TOF (Waters).

## S4. Clone assay

Translation of control peptide 3.1B was performed using a PURExpress™ Δ (aa, tRNA) *in vitro* protein synthesis kit (NEB) according to the manufacture's protocol, but with homemade solution A (50 mM HEPES-KOH, 2 mM ATP (Jena Bioscience), 2 mM GTP (Jena Bioscience), 1 mM CTP (Jena Bioscience), 1 mM UTP (Jena Bioscience), 20 mM creatine phosphate, 100 mM potassium acetate, 2 mM spermidine, 6 mM Magnesium Acetate, 1.5 mg/ml *E.Coli* tRNA (Roche), 14 µM DTT). Translation mixtures were prepared on ice by combining 0.78 µL solution A, 1.5 µL solution B, 0.5 µL each aminoacyl-tRNA (ClAc-

L-Trp-tRNA$^{fMet}{}_{CAU}$ and AcK-tRNA$^{Asn}{}_{CAU}$, prepared as described above S3.), 0.5 µL mRNA template (Template 2, 3.1B), 10 µM), 0.5 µL amino acid mixture (19 aa -Met), 0.72 µL water. The translation reaction mixture was incubated at 37 °C for 1 h. Following addition of 200 mM EDTA (pH 8.0), the translated mixture was reverse transcribed with M-MLV RTase, RNase H minus. The resulting mixture was first buffer exchanged into assay buffer (50 mM HEPES, 150 mM NaCl, 2 mM DTT, 0.1% Tween, pH 7.5) using a 1 mL sephadex column (G-25 fine, GE Healthcare) before the addition of 2x blocking buffer (50 mM HEPES, 150 mM NaCl, 2 mM DTT, 0.1% Tween, 0.2% (w/v) acetylated bovine serum albumin, pH 7.5). Following incubation with magnetic streptavidin bead (Life Technologies)-immobilised BRD3-BD1 (200 nM, 30 min), the beads were incubated with 5 M guanidine HCl (2x 20 min) at 0 °C before washing with ice-cold assay buffer (3x 1 min). Positive control was carried out without guanidine HCl treatment. PCR solution was added and the retained peptide-mRNA/DNA hybrids were eluted from the beads by heating (95 °C, 5 min). DNA recovery as a percentage of the input DNA was assessed by quantitative real-time PCR using primers T7g10M.F46 and CGS3an13.R22.

## S5. Bead-protein stability

Magnetic streptavidin bead-immobilised BRD3-BD2 (Life Technologies) (1 µM) was incubated with 0, 3, 5 or 8 M guanidine HCl (2x 20 min) at 0 °C. The beads were washed with ice-cold assay buffer (50 mM HEPES, 150 mM NaCl, 2 mM DTT, 0.1% Tween, pH 7.5) (3x 1 min). After heating to 95 °C for 5 min samples were analysed by SDS-PAGE on a 4–20% TruPAGE™ Precast gel (Sigma-Aldrich), and visualised by Quick Coomassie staining (Neo Biotech).

## S6. Peptide synthesis

### Bpa-P1 and Bpa-P2:

Linear precursors were assembled on solid-phase using standard Fmoc-protecting group strategy on an Intavis ResPep SLi automated synthesizer (CEM) using Rink Amide AM resin LL (0.05 mmol/g, Merck). All peptide couplings were performed with Fmoc-protected amino acids (5 equiv) in DMF, HATU (5 equiv) in DMF, and *N*,*N*-diisopropylethylamine (10 equiv) in NMP. Fmoc deprotection was carried out with 20% piperidine in DMF. *N*-terminal modification was performed manually by treating the resin-bound peptide with 0.18 M *N*-(chloroacetoxy)succinimide in DMF for 1 h.

Cleavage was achieved with a cocktail of trifluoroacetic acid (97.5%), water (2.5%) for 2 h. Note thiol scavengers were omitted to avoid the formation of side products due to reaction with the benzophenone. After diethyl ether precipitation, the peptide was lyophilised. The crude peptide was cyclised by incubating the linear peptide (< 1 mg/mL) in water with 10% acetonitrile and the pH adjusted to pH 7-8 using aqueous ammonia solution (0.25 M). The reaction mixture was shaken for 1 h, lyophilized and purified by HPLC using a reversed phase preparative C8 column (Agilent PrepHT

Zorbax 300SB-C8, 21.2x250 mm, 7 m) applying a flow rate of 8 mL/min and a linear gradient of 0 to 60% (v/v) solvent B for 40 min [solvent A: 98.92% (v/v) water, 0.08% (v/v) trifluoroacetic acid and 1% (v/v) acetonitrile; solvent B: 99.92% (v/v) acetonitrile and 0.08% (v/v) trifluoroacetic acid]. The purified cyclic peptides were analysed on an Agilent 1100 LC-MSD system (5-60% water/acetonitrile with 0.08% (v/v) trifluoroacetic acid over 8.5 min) (Supplementary Figure S9, Supplementary Table S2).

**Bpa-P1-FAM and Bpa-P2-FAM:**

Linear precursors were assembled on solid-phase using standard Fmoc-protecting group strategy on an Intavis ResPep SLi automated synthesizer (CEM) using Rink Amide AM resin LL (0.05 mmol/g, Merck). All peptide couplings were performed with Fmoc-protected amino acids (5 equiv) in DMF, HATU (5 equiv) in DMF, and *N*,*N*-diisopropylethylamine (10 equiv) in NMP. The side chain of cysteine was protected with monomethoxytrityl (Mmt), and side chain of *C*-terminal lysine was protected with 1-(4,4-dimethyl-2,6-dioxocyclohex-1-ylidene)-3-methylbutyl (ivDde). Fmoc deprotection was carried out with 20% piperidine in DMF. *N*-terminal modification was performed manually by treating the resin-bound peptide with 0.18 M *N*-(chloroacetoxy)succinimide in DMF for 1 h. This was followed by selective Mmt removal of the cysteine side chain by treatment with 1% trifluoroacetic acid in dichloromethane (8x 2 min). The resin was then washed with 2% *N*,*N*-diisopropylethylamine in DMF (2x 30 s) and on-resin cyclisation performed by incubation with 2% *N*,*N*-diisopropylethylamine in DMF for 16 h. Selective ivDde removal on the *C*-terminal lysine side chain was then achieved by treatment with 2% hydrazine in DMF (5x 10 min). Coupling with 5-FAM was performed by treatment with 5-FAM (4 equiv) in 500 µL DMSO:NMP (1:1), *N*,*N*-diisopropylethylamine (4 equiv), and oxyma pure (4 equiv) in NMP. After 3 min, DIC (4 equiv) was added and the mixture incubated for 30 min. The resin was washed with 20% piperidine in DMF (3x 1 min). Cleavage was achieved with a cocktail of trifluoroacetic acid (97.5%), water (2.5%) for 2 h. After diethyl ether precipitation and lyophilisation, the crude peptide was purified by HPLC using a reversed phase preparative C8 column (Agilent PrepHT Zorbax 300SB-C8, 21.2x250 mm, 7 m) applying a flow rate of 8 mL/min and a linear gradient of 0 to 60% (v/v) solvent B for 40 min [solvent A: 98.92% (v/v) water, 0.08% (v/v) trifluoroacetic acid, 1% (v/v) acetonitrile; solvent B: 99.92% (v/v) acetonitrile and 0.08% (v/v) trifluoroacetic acid]. The purified cyclic peptides were analysed on an Agilent 1100 LC-MSD system (5-100% water/acetonitrile with 0.08% (v/v) trifluoroacetic acid over 8.5 min) (Supplementary Figure S9, Supplementary Table S2).

**3.2B:**

Peptide previously prepared for use in ref. 2 was used for SPR experiments presented in Fig. 5E

## S7. Direct fluorescence anisotropy assay

BRD3-BD2 was diluted in assay buffer (50 mM HEPES, 150 mM NaCl, 0.05% Tween 20) to give a stock concentration of 400 µM. 2-fold serial dilutions were then made to give a 12-point dose-response curve. Peptides (Bpa-P1-FAM, Bpa-P2-FAM) were taken up in assay buffer with 0.1% DMSO to a concentration of 200 nM (100 nM final assay concentration).

Peptide (200 nM, 10 µL) and protein (variable concentration, 10 µL) were added to a 384-well plate (Greiner bio-one, 384 well, PS, Flat Bottom, Small Volume, Non-binding Microplate) and incubated at rt for 30 min. Experiments were performed in triplicate.

After 30 min, fluorescence anisotropy was measured with a CLARIOstar plate reader (BMG Labtech) using excitation at 482 nm and emissions at 530 nm at 25 °C. Background subtraction was performed using protein only controls. $K_D$ values were calculated using GraphPad Prism and a curve fitted using a one site - total fitting model.

## S8. Crosslinking

### Sample analysis by SDS PAGE:

Irradiated samples were analysed by SDS-PAGE on 4–20% Bis-Tris gels (mPAGE, Millipore). Experiments with Bpa-P1 and Bpa-P2 were visualised by Quick Coomassie staining (Neo Biotech) with gels imaged on a Chemidoc MP Imaging System (Biorad) and quantified by Fiji. Experiments with Bpa-P1-FAM and Bpa-P2-FAM were visualised by in gel fluorescence imaging using a Typhoon FLA 9500 (GE Healthcare).

### Sample analysis by LC-MS:

Irradiated samples with BRD3-BD2 were further analysed by LCMS. Briefly, denatured proteins were separated on a C4 BEH 1.7 µm, 1.0 x 100 mm UPLC column (Waters, UK) using a modified NanoAcquity (Waters, UK) to deliver a flow of approximately 50 µL/min. The column was developed over 20 minutes with a gradient of acetonitrile (2% v/v to 80% v/v) in 0.1% v/v formic acid. The analytical column outlet was directly interfaced via an electrospray ionisation source with a hybrid quadrupole time-of-flight mass spectrometer (Xevo G2, Waters, UK). Data was acquired over a m/z range of 300-3000, in positive ion mode with a cone voltage of 30 V. Scans were summed together manually and deconvoluted using MaxEnt1 (Masslynx, Waters, UK).

### Bpa-P1/P2-FAM and Lysate:

HEK293T cells were grown in DMEM supplemented with 10% FBS and penicillin/streptomycin at 37 °C and 5% $CO_2$. The cell line was obtained from and tested negative for mycoplasma by the Cell Services Technology Platform at the Francis Crick Institute. A mixture of FAM-labelled peptide (Bpa-P1-FAM or Bpa-P2-FAM; 4, 2 or 1 µM), recombinant BRD3-BD2 (1 µM) and lysate (HEK293T cells, 45 µg in 10 µl of lysate) in assay buffer were irradiated at 365 nm in a Longwave Ultraviolet Crosslinker (model CL-1000 L, UVP, analytikjena) for 1 h at 0 °C. Irradiated samples were analysed as described above. Control experiments were conducted without UV irradiation. Cell lysate was prepared as follows. HEK 293T cells were washed and resuspended in 1 x PBS then pelleted at 6,000 rpm for 2 min. The cell pellet was lysed with 50 mM Tris-HCl pH 7.5, 100 mM NaCl, 50 mM NaF, 1 % Triton X-100, 1 mM EDTA, 1 mM EGTA, and 1 mM DTT supplemented with protease inhibitor cocktail (Roche) and phosphatase inhibitors 2 & 3 (Sigma), and cell debris was removed by centrifugation at 13,000 rpm for 2 min.

## S9. Surface plasmon resonance (SPR)

Experiments were conducted on a Biacore S200 (GE Healthcare) and data analysed using the Biacore Insight Evaluation Software. Following the procedure in S8, biotinylated BRD3-BD2 (2 µM) was crosslinked with Bpa-P1 or Bpa-P2 (20 µM, Fig S8). Crosslinked BRD3-BD2, or control protein (irradiated in the presence of DMSO only) was immobilised on a CAP chip (GE Healthcare) to a target density of ~400 RU. Experiments were performed at 25 °C using all four lanes of the CAP chip in single cycle kinetics mode using 50 mM HEPES, 150 mM NaCl, 0.05% Tween-20, 0.1% DMSO, pH 7.5 as the running buffer. Between cycles the chip was regenerated following the manufacturer's protocol. Experiments were performed in triplicate.

## S10. Limited trypsinolysis

Purified BRD3-BD2 (20 µM) in presence or absence of cyclic peptide (Bpa-P1/Bpa-P2, 40 µM) was irradiated at 365 nm for 30 minutes. Following irradiation samples were incubated with 2 pmol of trypsin for 3 h at 37 °C. Samples were separated by SDS page and visualised by Coomassie staining to confirm digestion and analysed by LC-MS as in S7.

## S11. Mutagenesis

Mutagenesis of the plasmids expressing BRD3-BD2 were performed with the indicated primers using PfuTurbo DNAse Polymerase (Agilent) followed by DpnI treatment at 37 °C for 1 h. PCR product was then transformed into *Escherichia coli* DH5$\alpha$ High Efficiency (New England Biolabs) and plated on agar plates containing ampicillin (50 µg/mL). Mutations were confirmed by plasmid sequencing and protein expression was then carried out as described in the main text method "protein expression and purification". See Supplementary Table S1 for primers used in this study.

## Supplementary Figures

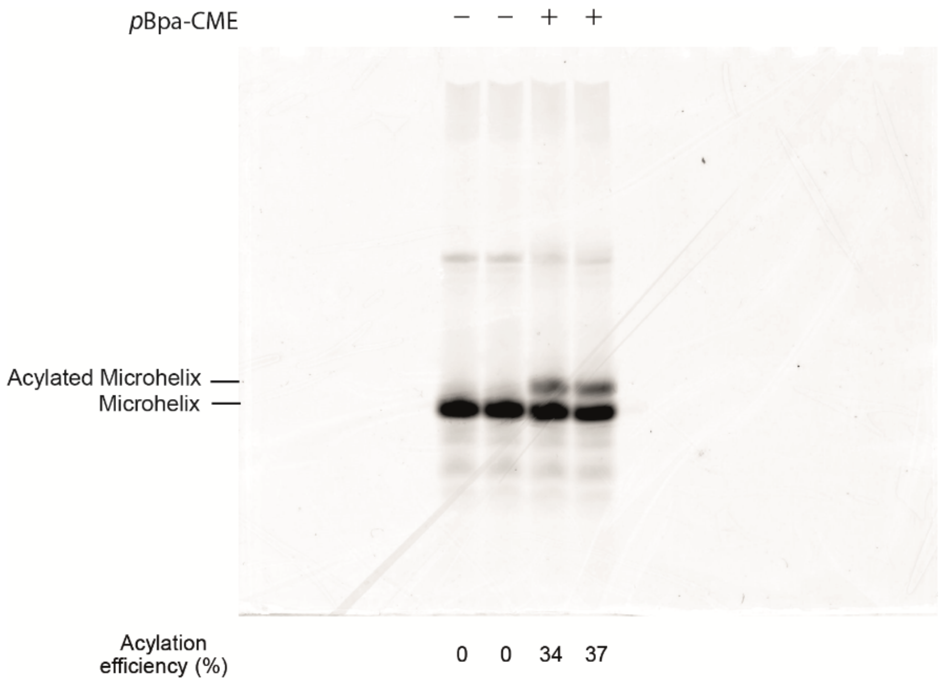


**Supplementary Figure S1.** Aminoacylation efficiency. eFx-mediated aminoacylation of microhelix RNA (FAM-MiHx_23b) with *p*Bpa-CME.

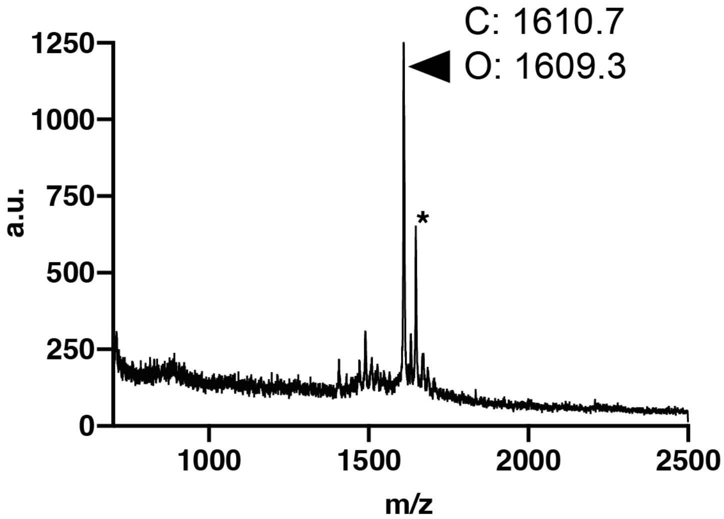


**Supplementary Figure S2.** Test translations. MALDI-TOF MS spectrum of translated cyclic peptide P0: (cyclic)-YKAF-*p*Bpa-GTVCGSGSGS. * corresponds to the non-cyclised peptide.

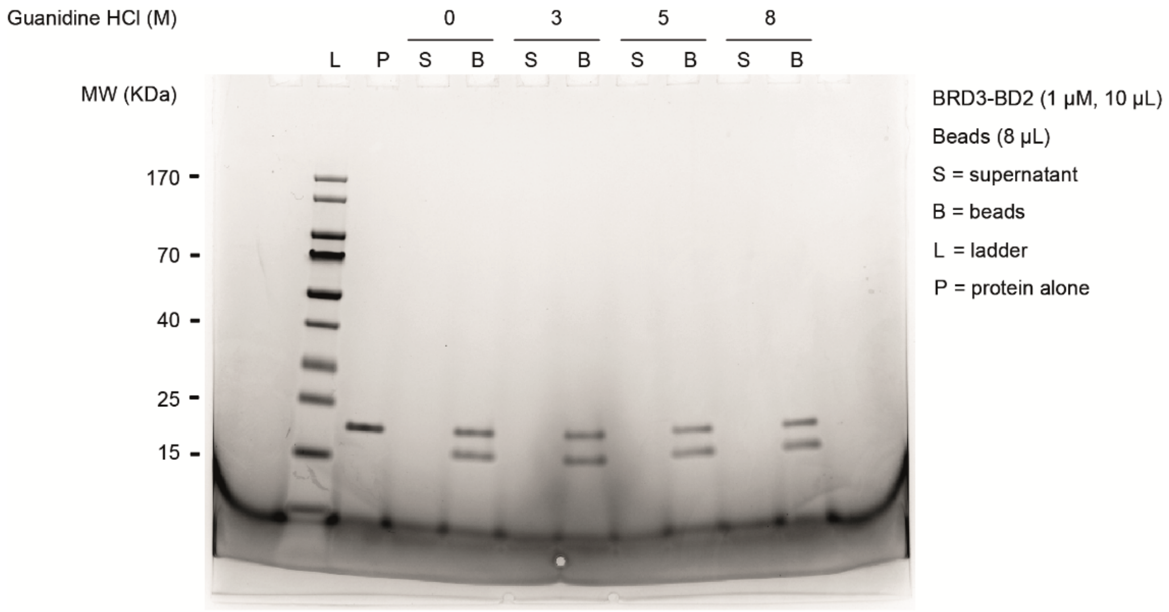


**Supplementary Figure S3.** Bead binding assessment. Amount of BRD3-BD2 retained on streptavidin beads when incubated with different concentrations of guanidinium chloride (0, 3, 5 or 8 M, 2x 20 min). Upper band corresponds to BRD3-BD2, lower band corresponds to streptavidin eluted from the beads.

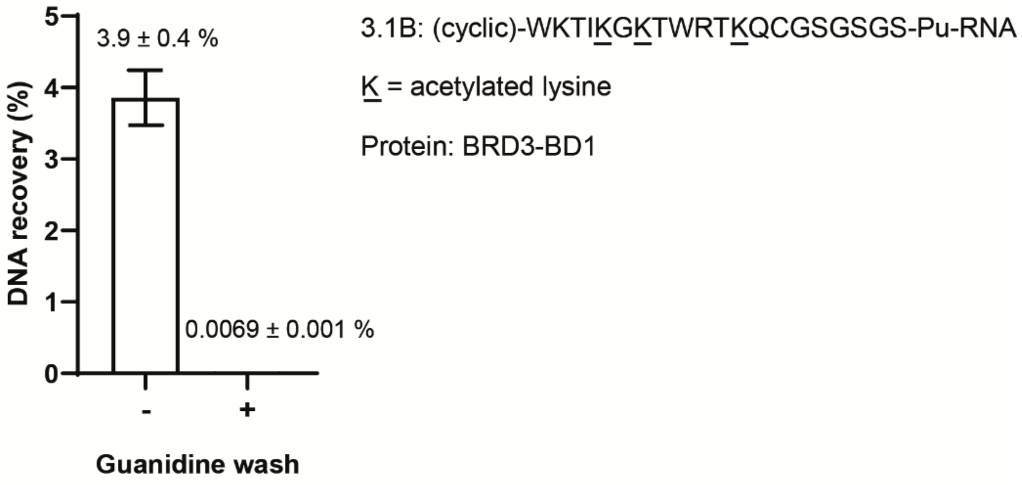


**Supplementary Figure S4.** Clone assays. Percentage of DNA recovered (Template 2, peptide 3.1B) after clone assay with/without incubation with guanidinium chloride (5 M, 2x 20 min), as assessed by quantitative real-time PCR relative to the input DNA.

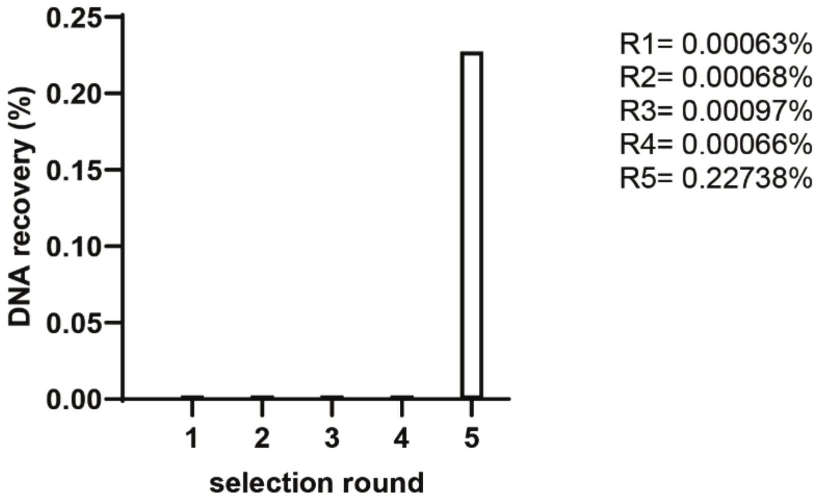


**Supplementary Figure S5.** Selection recoveries. Percentage of DNA recovered after each selection round, as assessed by quantitative real-time PCR relative to the input DNA library.

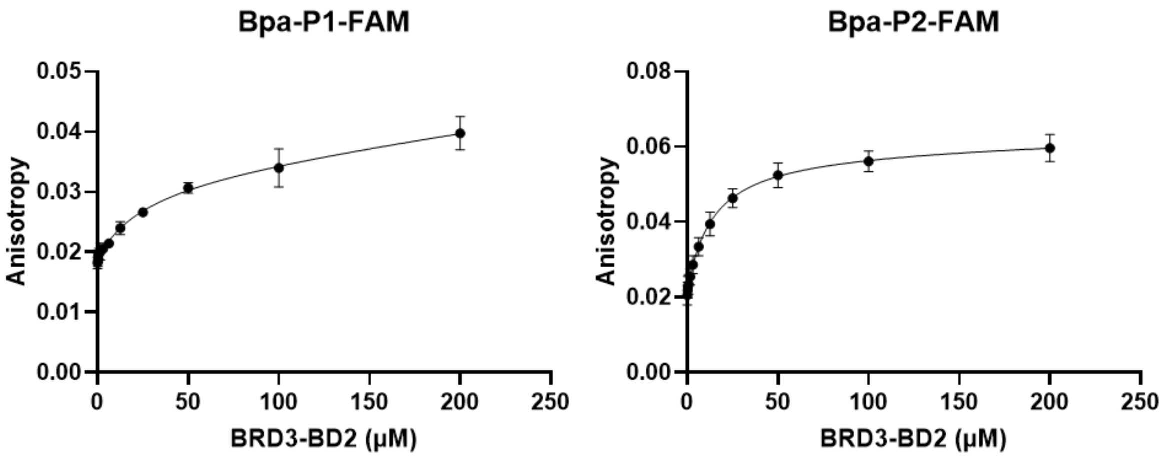


**Supplementary Figure S6.** Hit peptide binding affinities. Direct fluorescence anisotropy binding curves for FAM cyclic peptides Bpa-P1-FAM/Bpa-P2-FAM. Data represent the mean of triplicate experiments ± one standard deviation.

**a**

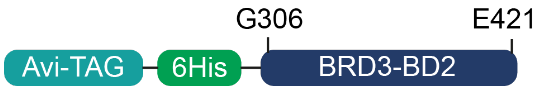


```
MSGLNDIFEAQKIEWHEGHHHHHHGSKGKLSEH
LRHCDSILREMLSKKHAAYAWPFYKPVDAEALE
LHDYHDIIKHPMDLSTVKRKMDSREYPDAQGFA
ADIRLMFSNCYKYNPPDHEVVAMARKLQDVFEM
RFAKMPDEPME
```

**b**

| | 2 pmol Trypsin 3 h 37 °C | | |
|---|---|---|---|
| Bpa-P2 (40 µM) | - | - | + |
| Bpa-P1 (40 µM) | - | + | - |
| BRD3-BD2 (20 µM) | + | + | + |
| Irradiation (30 min) | + | + | + |

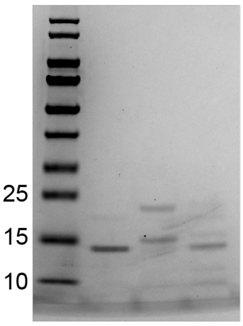


**c**

| Trypsinolysis 3 h at 37 °C | | | |
|---|---|---|---|
| No peptide | | Bpa-P1 | Bpa-P2 |
| M/Z | Sequence | M/Z | M/Z |
| 1005 | (R)LMFSNCYK(Y) | | |
| | | 1431 | |
| 1452 | | 1452 | |
| 2485 | (R)LMFSNCYKYNP PDHEVVAMAR(K) | | |
| 2694 | | 2694 | |
| 3036 | | 3036 | |
| 3206 | | 3206 | |
| 3293 | | 3293 | |
| 3335 | | 3335 | 3335 |

(R)LMFSNCYK(Y) → BRD3-BD2 381 - 390
(R)LMFSNCYKYNPPDHEVVAMAR(K) → BRD3-BD2 381 - 402

| Trypsinolysis 3 h at 37 °C | | | | | |
|---|---|---|---|---|---|
| No peptide | | Bpa-P1 | | Bpa-P2 | |
| M/Z | Sequence | M/Z | Sequence | M/Z | Sequence |
| 13500 | | | | 13500 | |
| 13686 | BRD3-BD2 (G306 - E421) | 13685 | | 13686 | |
| | | 15400 | | | |
| | | **15586** | BRD3-BD2 (G306 - E421) + Bpa-1 | 15554 | BRD3-BD2 (G306 - E421) + Bpa-2 |
| | | 16962 | | 16962 | |
| | | 18862 | | 17003 | |
| | | 18904 | | 18830 | |

**Supplementary Figure S7.** Limited trypsinolysis **a,** BRD3-BD2 construct sequence. **b,** trypsin digestion of BRD3-BD2 (20 µM) photocrosslinked in presence or absence of Bpa-1 or Bpa-2 (40 µM) for 3 h at 37 °C. **c,** peptides identified in intact-MS after trypsinolysis of BRD3-BD2 and BRD3-BD2 crosslinked with the different cyclic peptides.

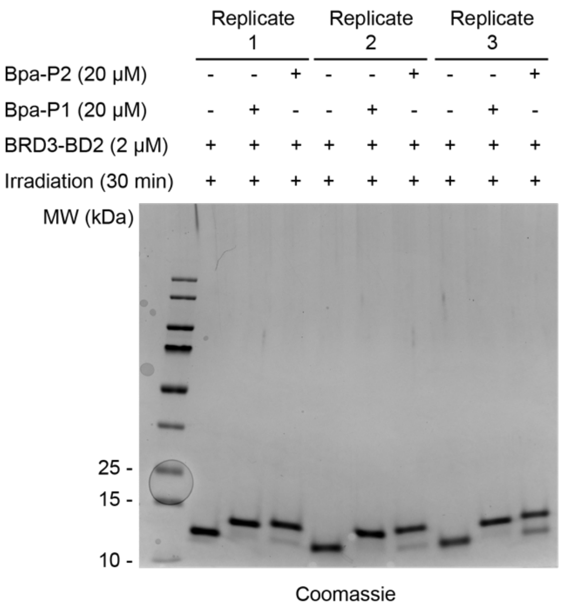


**Supplementary Figure S8.** Photocrosslinking of BRD3-BD2 (2 µM) by cyclic peptide (20 µM, Bpa-P1/Bpa-P2) after irradiation for 30 minutes prior to SPR shown in Fig 5e.

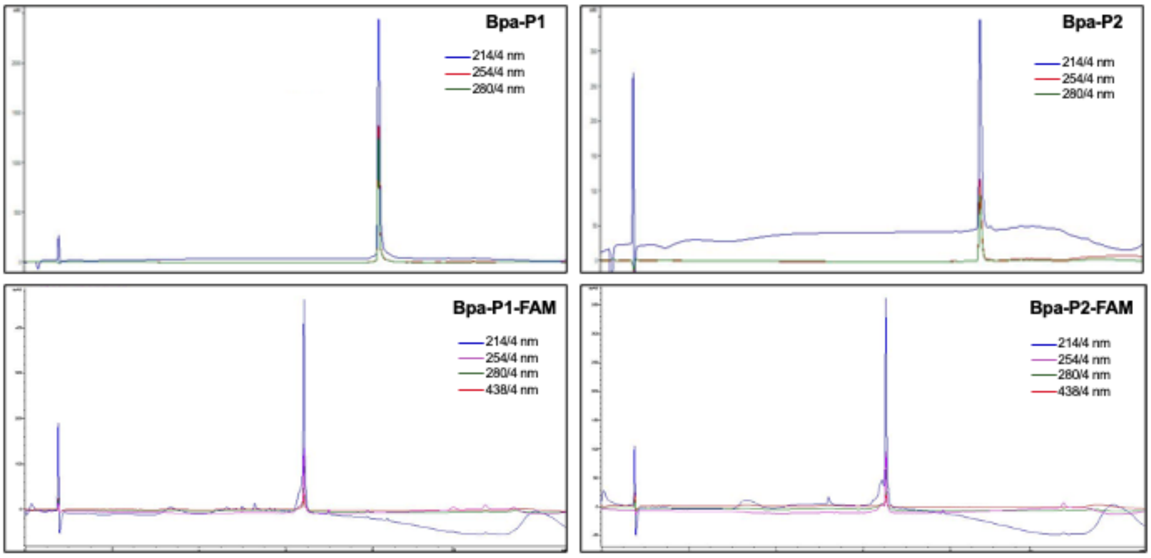


**Supplementary Figure S9.** LCMS chromatograms of purified peptides

**Full Gels:**

Complete gels shown partially in the main text and replicates used for quantification.

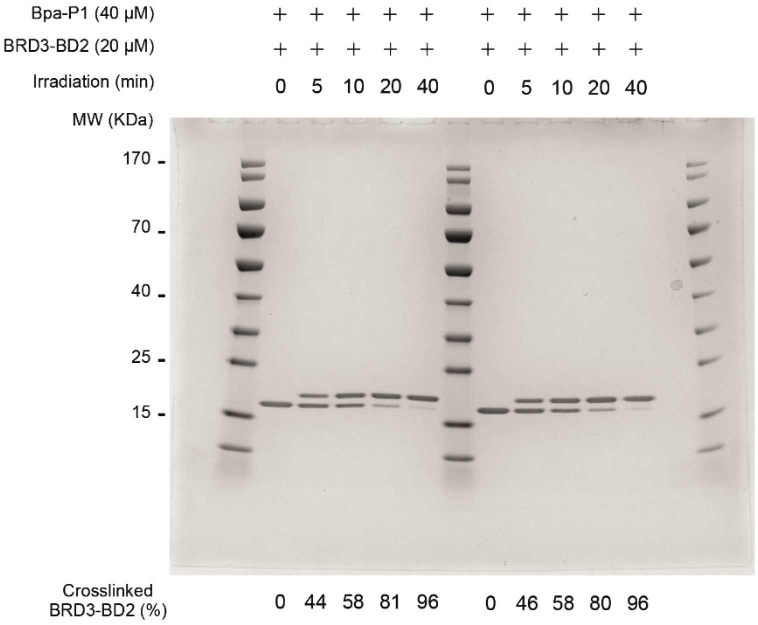


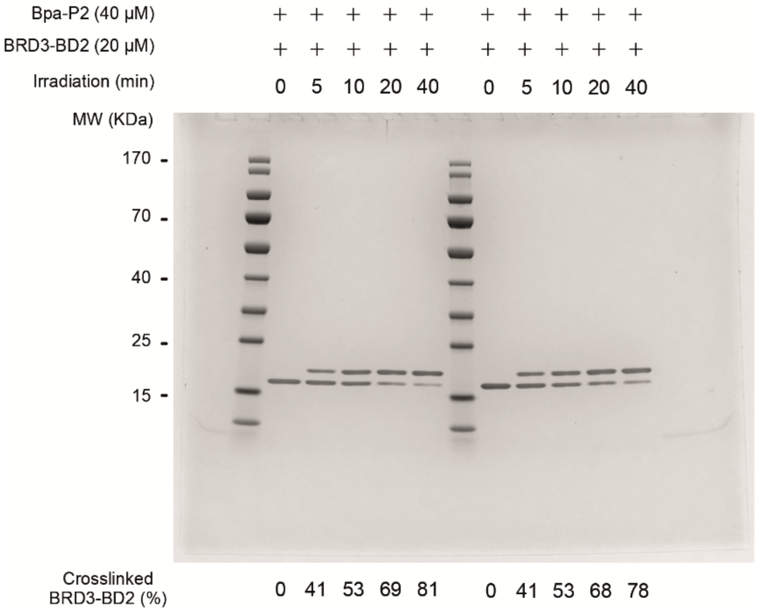


**Supplementary Figure S10.** Photocrosslinking of BRD3-BD2 (20 µM) by cyclic peptide (40 µM, Bpa-P1/Bpa-P2) after exposure to UV irradiation (365 nm) for variable amounts of time (0, 5, 10, 20, 40 min). Quantified data shown in Fig. 3a/b.

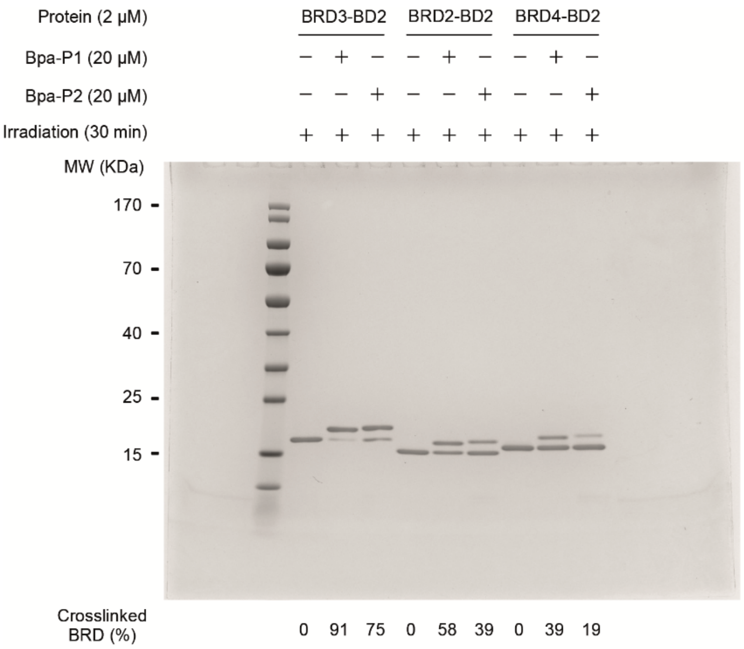
Protein (2 μM)
BRD3-BD2
BRD2-BD2
BRD4-BD2
Bpa-P1 (20 μM)
− + − − + − − + −
Bpa-P2 (20 μM)
− − + − − + − − +
Irradiation (30 min)
+ + + + + + + + +
MW (KDa)
170
70
40
25
15
Crosslinked BRD (%)
0 91 75 0 58 39 0 39 19

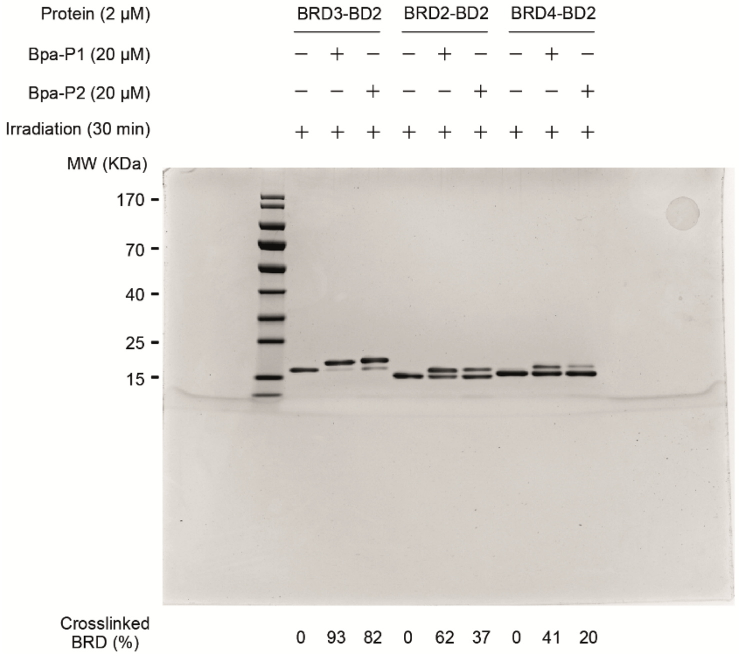
Protein (2 μM)
BRD3-BD2
BRD2-BD2
BRD4-BD2
Bpa-P1 (20 μM)
− + − − + − − + −
Bpa-P2 (20 μM)
− − + − − + − − +
Irradiation (30 min)
+ + + + + + + + +
MW (KDa)
170
70
40
25
15
Crosslinked BRD (%)
0 93 82 0 62 37 0 41 20

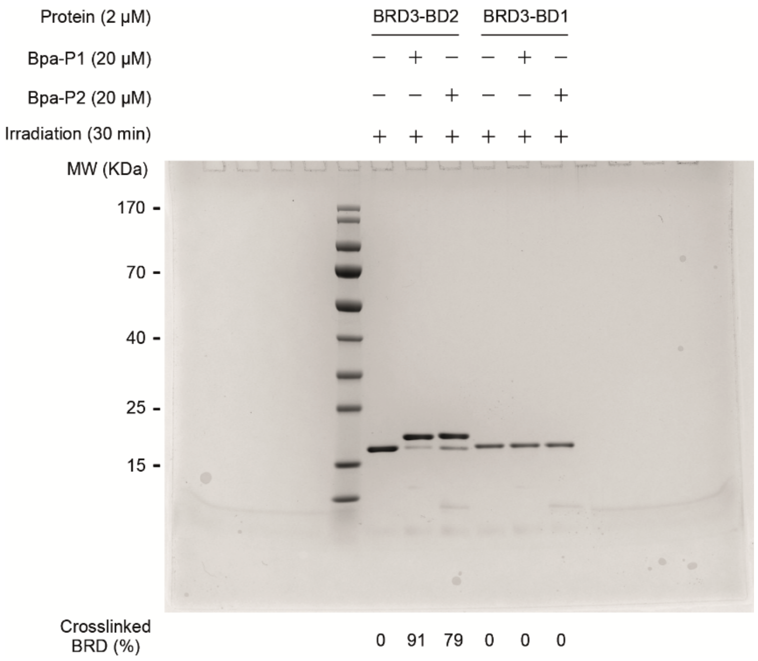


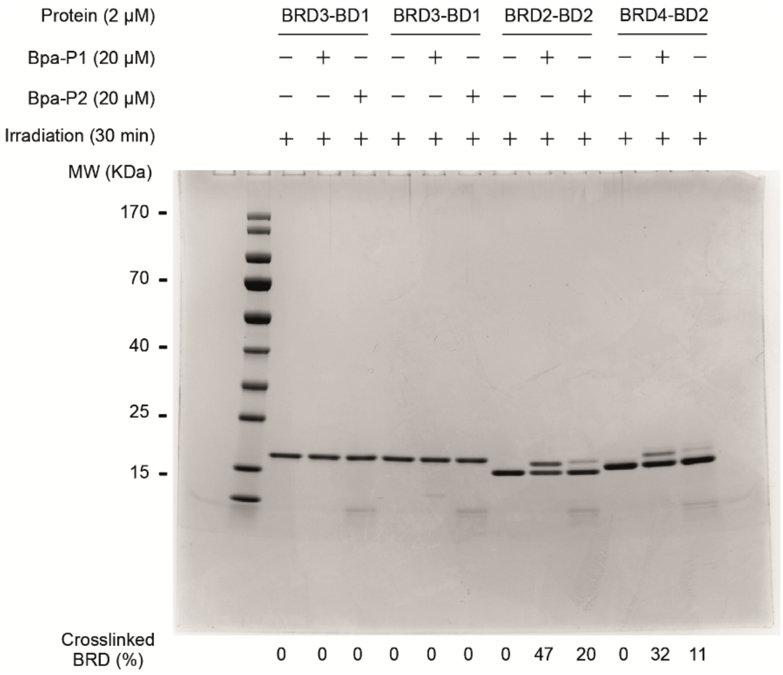


**Supplementary Figure S11.** Photocrosslinking of BRD3-BD1/BRD3-BD2/BRD2-BD2/BRD4-BD2 (2 μM) by cyclic peptide (20 μM, Bpa-P1/Bpa-P2) after exposure to UV irradiation (365 nm) for 30 min. Quantified data shown in Fig. 3c.

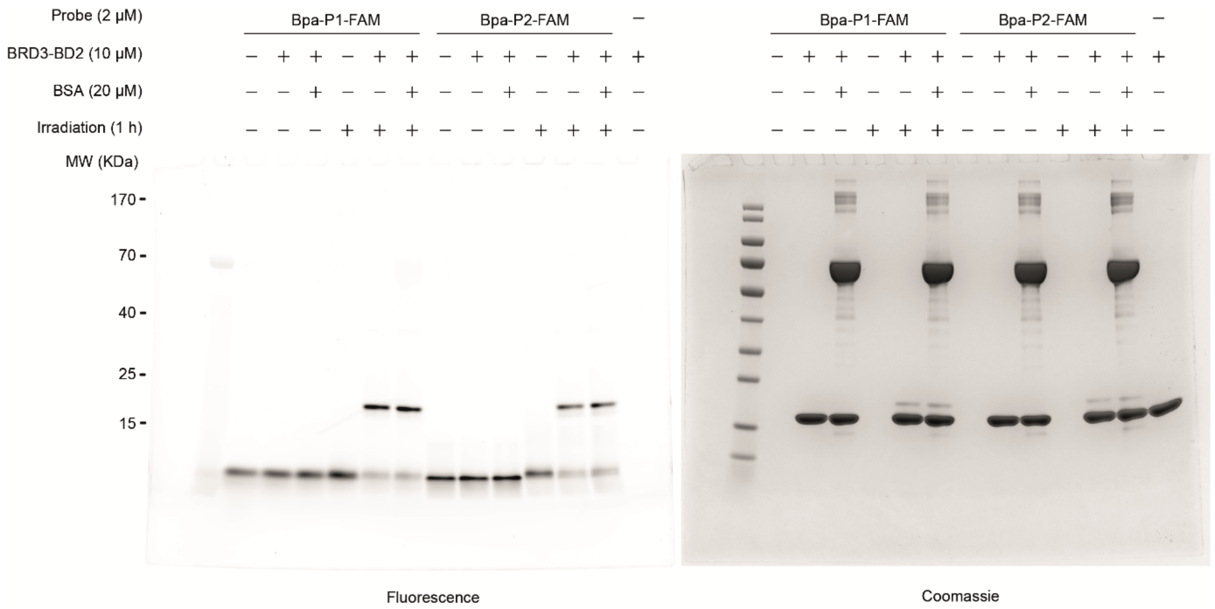


**Supplementary Figure S12.** Photoaffinity labelling of BRD3-BD2 (10 µM) with/without the presence of BSA (20 µM). BRD3-BD2 was preferentially labelled by FAM cyclic peptides (2 µM, Bpa-P1-FAM/Bpa-P2-FAM) after UV exposure (1 h). Control experiments without UV irradiation show no labelling. Partial gels shown in Fig 4a.

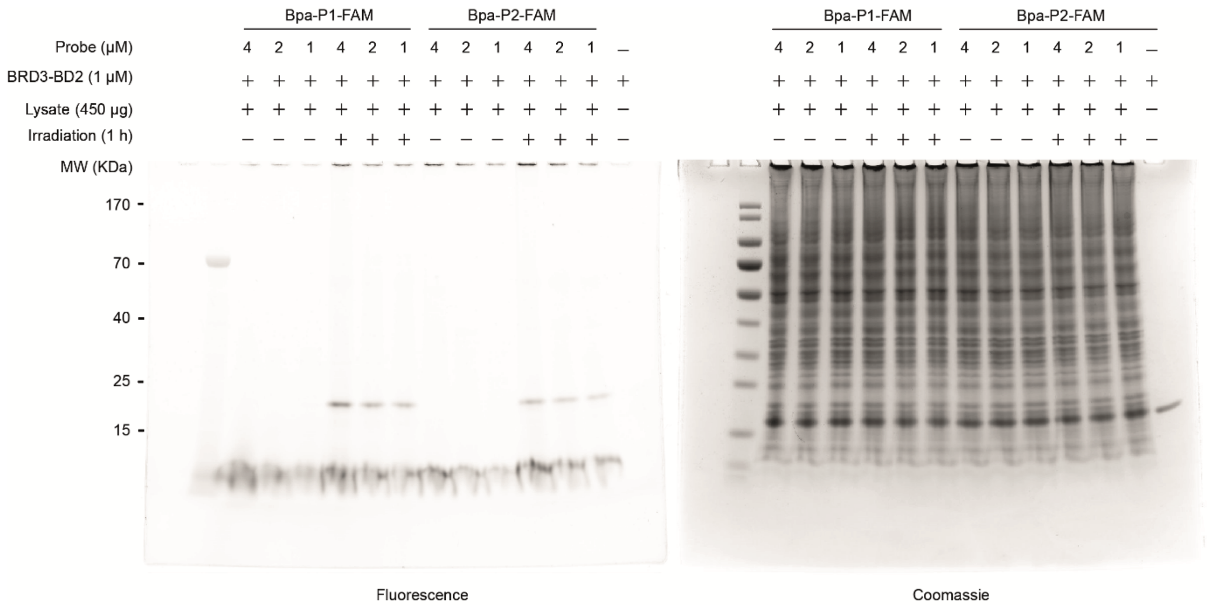


**Supplementary Figure S13.** Photoaffinity labelling of BRD3-BD2 (1 µM) spiked into cell lysate (450 µg, HEK293T cells). BRD3-BD2 was preferentially labelled by FAM cyclic peptides (4, 2 or 1 µM, Bpa-P1-FAM/Bpa-P2-FAM) after UV exposure (1 h). Control experiments without UV irradiation show no labelling. Partial gels shown in Fig 4b.

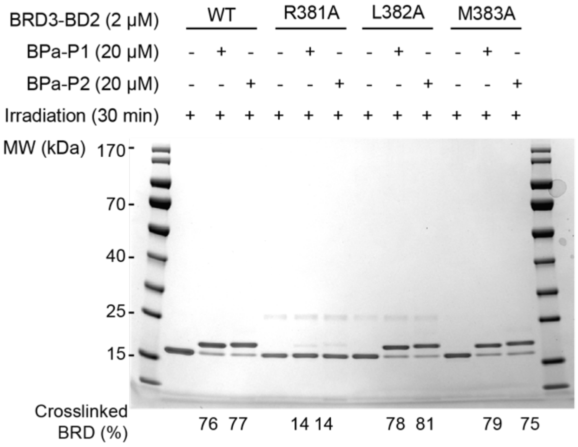
BRD3-BD2 (2 µM)
WT
R381A
L382A
M383A
BPa-P1 (20 µM)
BPa-P2 (20 µM)
Irradiation (30 min)
MW (kDa)
170
70
40
25
15
Crosslinked BRD (%)
76 77
14 14
78 81
79 75

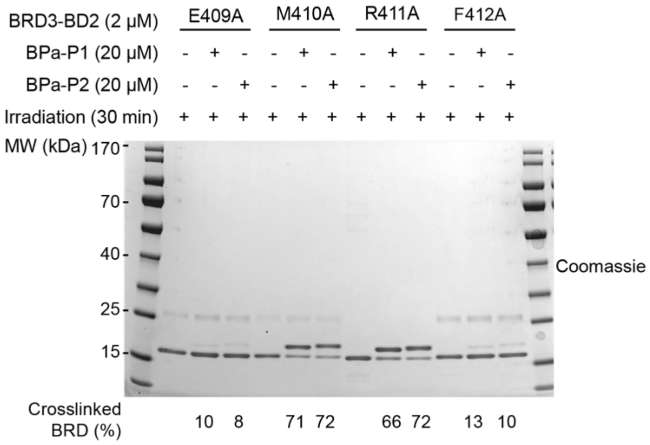
BRD3-BD2 (2 µM)
E409A
M410A
R411A
F412A
BPa-P1 (20 µM)
BPa-P2 (20 µM)
Irradiation (30 min)
MW (kDa)
170
70
40
25
15
Coomassie
Crosslinked BRD (%)
10 8
71 72
66 72
13 10

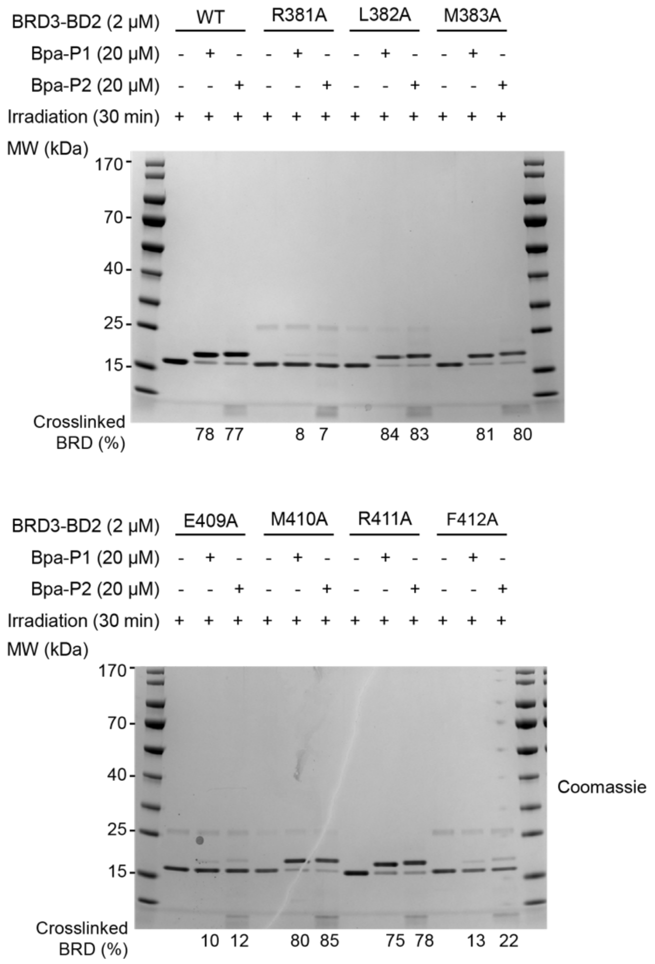


**Supplementary Figure S14.** Photocrosslinking of different BRD3-BD2 mutants (2 μM) by cyclic peptide (20 μM, Bpa-P1/Bpa-P2) after exposure to UV irradiation (365 nm) for 30 min. Quantified data shown in Fig. 5d.

## Supplementary Tables

**Supplementary Table S1.** List of oligonucleotides. N represents a 1:1 mixture of C and A, M represents a 1:1:1:1 mixture of all four DNA bases.

| Oligo ID | Sequence |
|---|---|
| T7g10M.F46 | TAATACGACTCACTATAGGGTTAACTTTAAGAAGGAGATATACATA |
| CGS3an13.R39 | TTTCCGCCCCCCGTCCTAGCTG |
| CGS3an13.R22 | TTTCCGCCCCCCGTCCTAGCTGCCGCTGCCGCTGCCGCA |
| Primer 1 (Template P0, MKAFMGTVCGSGSGS) | GCCGCTGCCGCTGCCGCACACGGTGCCCATAAACGCTTTCATATGTATATCTCCTTCTT |
| Primer 2 (Template 2, 3.1B, MKTIMGMTWRTMQC GSGSGS) | CCGCTGCCGCTGCCGCACTGCATGGTGCGCCAGGTCATGCCCATAATGGTTTTCATATGT ATATCTCCTTCTT |
| stdlib_NNK6_R63 | GCTGCCGCTGCCGCTGCCGCAMNNMNNMNNMNNMNNMNNCATATGTATATCTCCT TCTTAAAG |
| stdlib_NNK7_R66 | GCTGCCGCTGCCGCTGCCGCAMNNMNNMNNMNNMNNMNNMNNCATATGTATATC TCCTTCTTAAAG |
| stdlib_NNK8_R69 | GCTGCCGCTGCCGCTGCCGCAMNNMNNMNNMNNMNNMNNMNNMNNCATATGTA TATCTCCTTCTTAAAG |
| stdlib_NNK9_R72 | GCTGCCGCTGCCGCTGCCGCAMNNMNNMNNMNNMNNMNNMNNMNNMNNCATA TGTATATCTCCTTCTTAAAG |
| stdlib_NNK10_R75 | GCTGCCGCTGCCGCTGCCGCAMNNMNNMNNMNNMNNMNNMNNMNN MNNMNNCATATGTATATCTCCTTCTTAAAG |
| stdlib_NNK11_R78 | GCTGCCGCTGCCGCTGCCGCAMNNMNNMNNMNNMNNMNNMNNMNN MNNMNNMNNCATATGTATATCTCCTTCTTAAAG |
| stdlib_NNK12_R81 | GCTGCCGCTGCCGCTGCCGCAMNNMNNMNNMNNMNNMNNMNNMNN MNNMNNMNNMNNCATATGTATATCTCCTTCTTAAAG |
| FAM-MiHx_23b | 56-FAM/rArGrGrCrUrCrUrGrUrUrCrGrCrArGrArGrCrCrGrCrCrA |
| R381A_Fw | GGGCTTTGCTGCTGATATCGCGTTAATGTTCTCGAATTG |
| R381A-Rev | CAATTCGAGAACATTAACGCGATATCAGCAGCAAAGCCC |
| L382A_Fw | GGCTTTGCTGCTGATATCCGGGCAATGTTCTCGAATTGTTATAAGTAC |
| L382A_Rev | GTACTTATAACAATTCGAGAACATTGCCCGGATATCAGCAGCAAAGCC |
| M383A_Fw | GCTGCTGATATCCGGTTAGCGTTCTCGAATTGTTATAAG |
| M383A_Rev | CTTATAACAATTCGAGAACGCTAACCGGATATCAGCAGC |
| E409A_Fw | GCCAGGAAGCTCCAGGATGTGTTTGCGATGAGGTTTGCCAAGATGC |

| | |
|---|---|
| E409A_Rev | GCATCTTGGCAAACCTCATCGCAAACACATCCTGGAGCTTCCTGGC |
| M410A_Fw | GCTCCAGGATGTGTTTGAGGCGAGGTTTGCCAAGATGCCC |
| M410A_Rev | GGGCATCTTGGCAAACCTCGCCTCAAACACATCCTGGAGC |
| R411A_Fw | GCTCCAGGATGTGTTTGAGATGGCGTTTGCCAAGATGCCCG |
| R411A_Rev | CGGGCATCTTGGCAAACGCCATCTCAAACACATCCTGGAGC |
| R412A_Fw | GTGTTTGAGATGAGGGCTGCCAAGATGCCCGATG |
| R412A_Rev | CATCGGGCATCTTGGCAGCCCTCATCTCAAACAC |

**Supplementary Table S2.** LCMS data for cyclic peptides. The *m/z* ratios show the $[M+2H]^{2+}$ species.

| Peptide | *m/z* found | *m/z* calcd |
|---|---|---|
| Bpa-P1 | 950.9 | 950.9 |
| Bpa-P2 | 934.8 | 934.9 |
| Bpa-P1-FAM | 1193.7 | 1193.5 |
| Bpa-P2-FAM | 1177.7 | 1177.5 |

## Supplementary References


1 Y. Goto, T. Katoh, H. Suga, *Nat. Protoc.,* 2011, **6**, 779–790.

2 K. Patel, L. J. Walport, J. L. Walshe, P. D. Solomon, J. K. K. Low, D. H. Tran, K. S. Mouradian, A. P. G. Silva, L. Wilkinson-White, A. Norman, C. Franck, J. M. Matthews, J. M. Guss, R. J. Payne, T. Passioura, H. Suga, J. P. Mackay, *PNAS,* 2020, **117**, 26728–26738.
