# Supplementary material for: Identification of photocrosslinking peptide ligands by mRNA display
Source: Commun Chem. 2023 May 31;6:103. doi: 10.1038/s42004-023-00898-2 (PMC10232439; doi:10.1038/s42004-023-00898-2)
Supplement: Supplementary file 2 — Description of Additional Supplementary Files [file 42004_2023_898_MOESM2_ESM.pdf]

Description of Additional Supplementary File

File name: Supplementary Data 1

Description: Illumina sequencing data for recovered libraries from each round of XL-RaPID screening.
